# Bridging neurology and psychiatry: establishing Latin America’s first dedicated Neuropsychiatry Ward at IPq-HCFMUSP

**DOI:** 10.3389/fpsyt.2025.1621800

**Published:** 2025-07-31

**Authors:** Daniele Costa Rachid Lacerda, Lucas D’Andrea Pereira Sousa, Maruan Obeid, Livia Souza Santos, Luis Antonio Bozutti, Renato Luiz Marchetti

**Affiliations:** Institute of Psychiatry, Department of Psychiatry, Hospital das Clínicas, Faculty of Medicine, University of São Paulo, São Paulo, Brazil

**Keywords:** neuropsychiatry, integrated health care, research, medical education, epilepsy

## Abstract

**Background:**

The convergence of neurology and psychiatry has become critical for managing disorders with overlapping neurological and psychiatric features. To fill a service gap in Latin America, Brazil’s first Neuropsychiatry Ward (ENPQ) opened at the Institute of Psychiatry, Hospital das Clínicas, University of São Paulo in December 2024. This study describes the ward’s rationale, structure, and early clinical outcomes.

**Methods:**

We performed a retrospective descriptive analysis of all 26 adult admissions to the ten-bed ENPQ from 1 December 2024 to 11 April 2025. We extracted age, sex, length of stay (LOS), primary diagnosis, and 30-day readmission from electronic records and computed descriptive statistics.

**Results:**

Patients had a mean age of 48.0 ± 15.4 years (69.2% female), with one-third aged ≥ 60 years. Epilepsy was the most common primary diagnosis (46.2%), followed by Bipolar affective disorder, Depressive disorder and Schizophrenia (7.7% each). The mean LOS was 26.1 days, and only two patients (7.6%) were readmitted within 30 days—a rate lower than those reported for both general psychiatric wards (≈16%) and tertiary neurology wards (≈12.5%).

**Conclusions:**

In this study, an integrated neuropsychiatry ward demonstrated feasibility in a public quaternary hospital and yielded a lower 30-day readmission rate than non-integrated settings, despite a complex referral population and a LOS comparable to standard care. These findings support further prospective, controlled studies to confirm the benefits of integrated neurologic and psychiatric management.

## 1 Introduction

The need for an integrated approach between neurology and psychiatry becomes increasingly evident given the limitations of traditional models in providing satisfactory clinical solutions to complex disorders (1). Historically, these specialties emerged from a common matrix in the 19th century, particularly influenced by thinkers such as Wilhelm Griesinger, who advocated that mental illnesses were manifestations of brain disorders (2). However, throughout the 20th century, sociocultural and scientific factors favored the separation between neurology and psychiatry, resulting in distinct clinical and academic pathways (2).

The late 20th century witnessed a conceptual transformation leading to a broader understanding of neuropsychiatry (3). This theory emphasized that many psychiatric symptoms are best understood within the context of brain pathology, even when overt neurological signs are not readily apparent. In addition to, in recent decades, propelled by advances in neuroscience, neuroimaging and neurogenetics, neuropsychiatry has experienced a true rebirth (2, 5). This field, currently recognized as essential for the understanding and management of various behavioral and cognitive conditions, seeks to transcend traditional disciplinary boundaries by promoting a unified view of brain and mind (5). Recent work demonstrates that disorders once thought to be purely psychiatric actually harbor measurable neurobiological alterations, underscoring the imperative for integrated clinical care (1). At the macroscopic level, structural and microstructural neuroimaging has identified volumetric changes in limbic and prefrontal regions in both temporal lobe epilepsy with psychotic features and autoimmune encephalitis, pointing to synaptic and network-level dysfunctions that bridge neurological and psychiatric domains (6, 7). Complementing these imaging findings, molecular investigations reveal a sustained neuroinflammatory milieu in such conditions. Pro-inflammatory cytokines — including interleukin-1β (IL-1β), interleukin-6 (IL-6), and tumor necrosis factor-α (TNF-α) — are significantly elevated in the serum and cerebrospinal fluid of patients with temporal lobe epilepsy and related syndromes (7). In particular, IL-1β amplifies excitotoxicity by potentiating NMDA-receptor–mediated glutamatergic transmission and diminishing GABA-A currents, while IL-6, released by activated astrocytes and microglia, remains raised for up to 24 hours after seizure, reflecting persistent inflammation (7, 8). This inflammatory cascade also contributes to blood–brain barrier breakdown (BBB), facilitating further immune cell infiltration and neuroimmune activation (6). In parallel, autoantibody-mediated mechanisms disrupt synaptic integrity in autoimmune encephalitis. Antibodies against the NMDA receptor’s GluN1 subunit induce receptor internalization and hypofunction, leading to psychiatric symptoms, memory impairment, and seizures (9). Such convergent evidence — from cytokine profiles and barrier dysfunction to receptor-targeting autoantibodies — provides mechanistic insight into how immune and structural perturbations underlie overlapping neurological and psychiatric manifestations. Elevated Cerebrospinal Fluid/Serum albumin ratios in epilepsy patients further corroborate BBB leakage and correlate with seizure frequency (10). Beyond anti-NMDAR antibodies, autoantibodies against LGI1 disrupt the LGI1–ADAM22/23 complex essential for AMPA receptor clustering, reducing postsynaptic AMPAR density and impairing synaptic plasticity (11, 12). These processes converge on microglial activation: cytokine-mediated and antibody-driven insults trigger microglial release of additional pro-inflammatory mediators (e.g., IL-12, CCL2), which perpetuate synaptic pruning and neuronal dysfunction (13). Together, the convergence of neuroinflammatory signals, matrix metalloproteinase–induced blood–brain barrier breakdown, and various autoantibody‐driven synaptic disturbances reveals a complex pathophysiology that unites neurological and psychiatric disease processes.

The establishment of specialized neuropsychiatry wards reflects this contemporary movement toward reintegration. Such units provide hospitalizations managed by multidisciplinary teams equipped to address severe and complex neuropsychiatric disorders, in which organic and psychosocial factors are deeply intertwined (5). Internationally, examples of these integrated models include the Division of Neuropsychiatry and Neuromodulation at Massachusetts General Hospital (14), the Division of Neuropsychiatry at Brigham and Women’s Hospital (15), and the North Staffordshire Neuropsychiatry Service in the United Kingdom (16) demonstrating that the need for specialized services extends beyond major academic centers and is equally relevant in regional settings.

The ENPQ admits adults with severe and refractory presentations in which neurological and psychiatric elements are deeply intertwined. Examples include refractory epilepsy complicated by mood disturbances or psychosis (e.g., temporal lobe epilepsy–associated psychotic disorder), autoimmune encephalitis presenting with catatonia or acute psychosis, neurodegenerative diseases such as Parkinson’s disease or Huntington’s disease with comorbid depression, anxiety or cognitive impairment, functional neurological symptom disorders co-occurring with severe affective dysregulation, traumatic brain injury sequelae featuring mood lability and cognitive deficits, metabolic or genetic syndromes manifesting combined neurological and psychiatric features, and psychotropic-induced neurotoxicities requiring specialized monitoring. Such cases benefit from simultaneous neurologic evaluation (e.g., EEG, imaging) and psychiatric management (e.g., psychotropic adjustments, behavioral interventions), underscoring the need for an integrated ward.

To deliver integrated care, the neuropsychiatry ward assembles a truly multidisciplinary team: neurologists with expertise in EEG monitoring and management of seizure disorders; psychiatrists; neuropsychologists conducting detailed cognitive and functional evaluations; specialized nursing staff trained in neuropsychiatric risk assessment and monitoring; EEG and neurophysiology technicians enabling continuous video-EEG when indicated; clinical pharmacists optimizing complex pharmacotherapeutic regimens; social workers coordinating discharge planning and community reintegration; and, where needed, immunologists or rheumatologists collaborating on suspected autoimmune syndromes. This composition ensures that both organic brain disorders and psychiatric manifestations are addressed concurrently, with shared decision-making and joint care planning (11).

In Brazil, following this international trend, the Institute of Psychiatry of the Hospital das Clínicas, Faculty of Medicine, University of São Paulo (IPq-HCFMUSP) inaugurated the Neuropsychiatry Ward (ENPQ) in December 2024. This unit was designed to provide short-term multidisciplinary hospitalizations for adults with severe and refractory neuropsychiatric disorders. Its structure and philosophy represent a strategic advancement not only in clinical care but also in the training of new specialists and in strengthening translational research by fostering the intersection between neurology, psychiatry, and neuroscience (1–5).

This article aims to describe the conceptual foundation, organizational process, and initial operating characteristics of the ENPQ at IPq-HCFMUSP. Additionally, it presents an exploratory analysis of the sociodemographic and clinical data of patients admitted since the ward’s inauguration.

## 2 Materials and methods

### 2.1 Study design and setting

This retrospective, observational descriptive study was conducted at the ENPQ of IPq-HCFMUSP. The unit, opened in December 2024, comprises ten beds (including one equipped for video-EEG monitoring and another designated for semi-intensive care) and provides short, multidisciplinary hospitalizations for adults with complex neuropsychiatric conditions. We analyzed all admissions (n = 26) between 1 December 2024 and 11 April 2025.

### 2.2 Admission criteria

Screening is performed by attending psychiatrists. Eligible individuals are those aged ≥ 18 years who meet at least one of the following criteria: decompensated neuropsychiatric disorder, need for immediate hospital-based evaluation or intervention and selected cases of particular academic or training interest that align with the unit’s clinical, educational, and research objectives. To operationalize the cases of particular academic, all potential referrals under this category are evaluated by a neuropsychiatrist using a standardized checklist addressing: (a) clinical complexity (presence of overlapping neurological and psychiatric features requiring integrated management, e.g., temporal lobe epilepsy with refractory mood or psychotic symptoms, suspected autoimmune encephalitis with behavioral manifestations, or neurodegenerative disease plus comorbid affective dysregulation); (b) novelty or rarity (unusual etiologies or presentations that may yield insights for translational inquiry, such as paraneoplastic syndromes or rare genetic conditions with neuropsychiatric phenotypes); (c) potential contribution to ongoing or planned research protocols (feasibility of prospective data collection in the REDCap database, availability of informed consent for additional assessments or biomarker studies, and alignment with predefined hypotheses); (d) educational value (cases that illustrate diagnostic and therapeutic challenges valuable for fellows’ learning, such as titration strategies in complex polypharmacy).

### 2.3 Data collection

We extracted sociodemographic variables (age range, gender), service‐use metrics (length of stay), clinical characteristics (primary neuropsychiatric diagnosis), and clinical outcome measures (30-day readmission rate) from the electronic medical records of the 26 patients. Trained reviewers collected and entered all information into a standardized spreadsheet, ensuring consistency and reliability of the dataset.

### 2.4 Statistical analysis

Continuous variables were summarized by means ± standard deviation; categorical variables by absolute and relative frequencies. Analyses were performed using R statistical software (v4.3.2), and the results are presented in descriptive tables and graphs.

### 2.5 Ethics

Institutional approval for this retrospective observational study was obtained from the Research Ethics Committee (Comissão de Ética em Pesquisa) of Hospital das Clínicas, Faculty of Medicine, University of São Paulo (CAPPesq/HCFMUSP). All procedures adhered to the ethical principles of the Declaration of Helsinki (17) and to Brazilian National Health Council Resolution CNS 466/2012, which governs research with human subjects in Brazil (18). Given the use of de-identified, routinely collected clinical data, the requirement for informed consent was formally waived by the committee. Data confidentiality and patient privacy were maintained according to institutional policies and in compliance with the Brazilian General Data Protection Law (Lei Geral de Proteção de Dados Pessoais, Lei No. 13.709/2018) (19). The study was conducted in accordance with applicable national and institutional guidelines for research involving human participants, and the CAPPesq/HCFMUSP operates in alignment with CONEP/CNS regulations for ethical review (20).

## 3 Results

### 3.1 Patient characteristics

Between December 2024 and April 2025, 26 patients were admitted to the ENPQ. As shown in **Table 1**, the mean ± SD age was 48.0 ± 15.4 years, and 30.8% were aged ≥ 60 years. Most patients were female (69.2%). The mean length of stay was 26.1 days. Only two patients were readmitted within 30 days, yielding a 7.6% readmission rate - below the 10–20% reported for Brazilian general-hospital psychiatric wards (5).

**Table 1:** Sociodemographic characteristics of patients admitted to the ENPQ (n = 26).

Variable	n	%
Gender		
Male	8	30.80%
Female	18	69.20%
Age Range		
18–29	5	19.20%
30–39	3	11.50%
40–49	6	23.10%
50–59	4	15.40%
≥ 60	8	30.80%

### 3.2 Diagnoses

**Figure 1:**
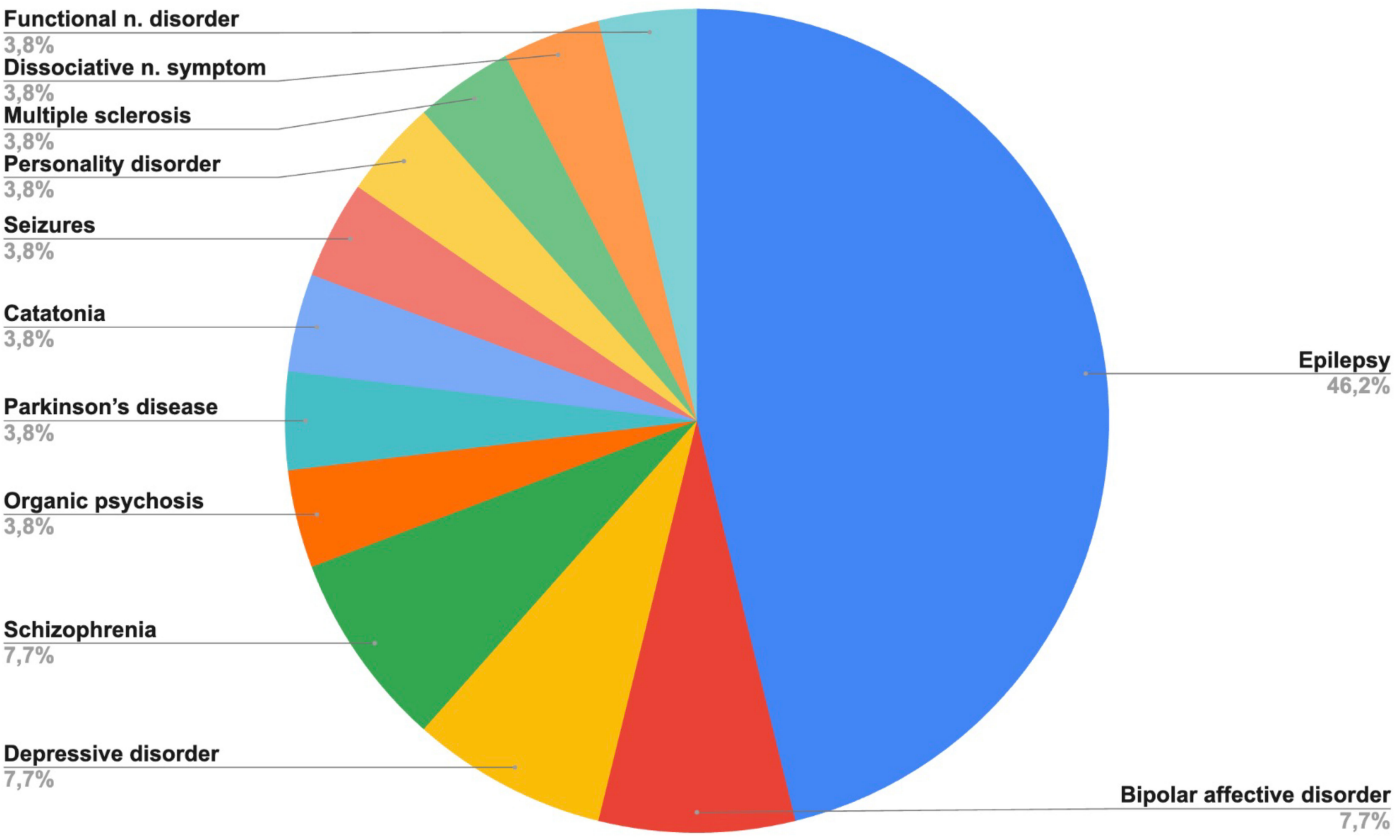
Distribution of primary neuropsychiatric diagnoses among patients admitted to the ENPQ between December 2024 and April 2025. “Functional n. disorder,” i.e., Functional neurological disorder; “Dissociative n. symptom,” i.e., Dissociative neurological symptom.

Epilepsy accounted for 46.2% of cases, followed by Bipolar affective disorder, Depressive disorder and Schizophrenia (7.7% each). **Table 2**; **Figure 1** illustrate the diagnostic distribution, highlighting the diversity of presentations managed at the ENPQ.

**Table 2:** Primary neuropsychiatric diagnoses of patients admitted to the ENPQ (n = 26).

Diagnosis	n	%
Epilepsy	12	46.2%
Bipolar affective disorder	2	7.7%
Depressive disorder	2	7.7%
Schizophrenia	2	7.7%
Organic psychosis	1	3.8%
Parkinson’s disease	1	3.8%
Catatonia	1	3.8%
Seizures	1	3.8%
Personality disorder	1	3.8%
Multiple sclerosis	1	3.8%
Dissociative neurological symptom	1	3.8%
Functional neurological disorder	1	3.8%

### 3.3 Clinical course

In our integrated ward, we assessed psychiatric status at admission and discharge using the Clinical Global Impression–Severity (CGI-S) and Clinical Global Impression–Improvement (CGI-I) scales, respectively. We defined “stabilization” of psychiatric symptoms as no more than a 10% increase on disorder-specific scales plus a CGI-I rating of “no change” to “minimally improved” at weekly interim reviews, and “improvement” as a CGI-I rating of “much improved” or better at discharge.

We systematically tracked core psychiatric features for each diagnostic group: in patients with epilepsy and seizures, we monitored interictal behavioral dysregulation and postictal confusion; for those with psychotic disorders or schizophrenia, we assessed the presence and severity of hallucinations, delusions, and disorganized thought; bipolar presentations were characterized by mood lability and overt manic symptoms; dissociative neurological symptom disorder cases were evaluated for non-epileptic seizure–like events and the degree of accompanying anxiety; organic mental disorders and delirium were marked by fluctuating attention and acute confusion; depressive disorders were monitored via changes in depressed mood, anhedonia, and psychomotor retardation; phobic anxiety disorder presentations focused on panic attacks and anticipatory anxiety; avoidant-restrictive food intake disorder cases were noted for restrictive eating behaviors and weight-related distress; personality disorder features comprised impulsivity and affective instability; and catatonia or neuroleptic malignant syndrome episodes were identified by immobility, mutism, and autonomic instability.

Neurological function was evaluated via objective measures tailored to each condition. Seizure frequency and severity for epilepsy and seizures were verified by nursing staff, with improvement expressed as percent reduction from baseline. Continuous video-EEG data were reviewed for decreases in interictal epileptiform discharges. Cognitive performance in mild and major neurocognitive disorders was screened using the Montreal Cognitive Assessment (MoCA) or Mini-Mental State Examination (MMSE). Motor signs in Parkinson’s disease were noted via clinician-rated bradykinesia and rigidity assessments, and sensorimotor deficits in multiple sclerosis and functional neurological symptom disorder were observed clinically. Level of consciousness and neuromuscular signs were recorded in organic or metabolic conditions (e.g., drug intoxication, neuroleptic malignant syndrome).

To ensure consistency, all assessments were performed within 48 hours of admission, repeated weekly, and again before discharge.

## 4 Discussion

### 4.1 Key findings

Data from the ENPQ’s first months reveal a predominantly female sample with a mean age of ≈ 48 years; one third were elderly. The mean length-of-stay was < 1 month, and the 30-day readmission rate was only 7.6%, suggesting effective clinical management. Epilepsy was the most prevalent diagnosis, reflecting the unit’s referral profile and its linkage to Projepsi (the IPq Epilepsy Program, structured as an outpatient service).

In interpreting our key outcomes, we compared the ENPQ’s metrics with published data from traditional, non-integrated inpatient settings. For example, the 30-day psychiatric readmission rate in general hospital psychiatric wards in Brazil and other settings is typically reported between approximately 10% and 20% (21). In contrast, our integrated neuropsychiatry ward observed a 7.6% 30-day readmission rate. Although differences in patient case-mix and referral patterns limit direct comparability, this lower readmission rate suggests that the simultaneous neurologic and psychiatric management model may help stabilize complex patients more effectively before discharge, potentially reducing early returns to hospital (22). Our mean LOS of 26.1 days is broadly similar to these figures. However, in standard psychiatric wards, patients with comorbid neurological conditions may require sequential referrals (first to psychiatry, then separately to neurology) or extended stays due to care fragmentation. In ENPQ, we manage both dimensions concurrently, which may explain why our LOS remains in line with expectations for severe cases, without prolongation despite higher clinical complexity.

In a large Brazilian tertiary neurology ward study including 2,606 admissions over an 11-year period, the mean patient age was 48.8 ± 18.0 years, 52.2% were female, the mean length of stay was 16.7 days, and the 30-day readmission rate was 12.5% (23). By contrast, our ENPQ cohort had a mean age of 48.0 ± 15.4 years (comparable in age), a higher proportion of female patients (69.2%), a longer mean length of stay of 26.1 days - reflecting the added complexity of integrated neuropsychiatric management - and a lower 30-day readmission rate of 7.6%. Although differences in case mix and referral pathways limit direct comparability, the longer stay at ENPQ likely corresponds to the need for simultaneous neurologic and psychiatric interventions, while the lower readmission rate suggests that the integrated model may achieve greater stabilization before discharge than traditional neurology or psychiatric wards operating separately.

We recognize that retrospective comparisons with literature benchmarks have inherent limitations, including differences in case severity, admission policies, and healthcare system factors. Ideally, future studies would enroll a matched cohort of patients admitted to separate psychiatry or neurology wards with similar neuropsychiatric profiles, using propensity-score matching or other methods, to compare LOS, readmission rates, symptom trajectories (using standardized scales), and patient-centered outcomes. Cost-effectiveness analyses should also model resource utilization in both settings. Such comparative data will more definitively quantify the added value of the integrated model.

### 4.2 Strengths and limitations

In our view, a key strength of the ENPQ model is the clearly defined, adequately staffed multidisciplinary team. For example, the ward operates with four dedicated neuropsychiatrists, two neurologists (including one with EEG monitoring expertise), one full-time neuropsychologist, one pharmacist, a social worker, and a nursing team providing 24/7 coverage (approximately two nurses per shift for the ten-bed unit). The structured team size and roles facilitate coordinated rounds, joint decision-making, and rapid adjustments of complex treatment plans, contributing to efficient, integrated care delivery. Another strength is the study’s focus on the psychiatry–neurology interface, addressing an area of recognized importance but limited prior service models in Latin America. By documenting initial outcomes from Brazil’s first dedicated neuropsychiatry ward, this work contributes concrete data to support the bridging of disciplines, as advocated in recent literature emphasizing the need to overcome traditional silos between neurology and psychiatry (1).

Its formal linkage with Projepsi and long-term integration with the Psychosocial Care Network (RAPS) constitute organizational innovations in Brazil, ensuring seamless transitions of care across all levels of complexity.

Preliminary cost-effectiveness analysis indicates that the observed reduction in 30-day readmission rates at ENPQ may help offset the initial investments required to establish and operate an integrated neuropsychiatric care model. By potentially decreasing rehospitalization rates, this approach could reduce overall healthcare expenditures related to inpatient psychiatric and neurological care, including costs associated with emergency interventions, prolonged hospital stays, and recurrent admissions. Demonstrating feasibility, acceptability, and initial clinical metrics in a public quaternary hospital highlights the potential for wider adoption of integrated care and informs training programs aimed at strengthening the neuropsychiatric workforce.

Among limitations, we note the persistent gaps in the literature regarding specific neurobiological substrates underlying complex neuropsychiatric syndromes. Although our exploratory data capture will eventually include biomarkers, neuroimaging, and neurophysiological measures, currently published evidence remains sparse and often derived from small case series or heterogeneous cohorts (24).

This scarcity constrains our ability to link clinical outcomes directly to underlying biological mechanisms in a standardized way. Furthermore, the retrospective design and small sample size limit generalizability; prospective, multimodal studies with larger cohorts are needed to elucidate pathophysiological substrates (e.g., network connectivity changes, neuroinflammatory markers) and to validate which integrated interventions most directly impact underlying biology. Acknowledging this gap underscores the need for future research integrating systematic biomarker collection with clinical metrics in neuropsychiatry.

### 4.3 Implications and future directions

Integrating systematic multimodal biomarker collection into the ENPQ workflow could deepen understanding of the biological substrates underlying complex neuropsychiatric syndromes. Future studies should embed protocols for fluid biomarkers (e.g., neuroinflammatory cytokines, neurofilament light) and advanced neuroimaging (e.g., network connectivity analyses via resting-state fMRI), correlating these measures with standardized clinical outcomes. Such efforts align with emerging trends emphasizing objective markers over solely subjective rating scales in neuropsychiatry research (25).

Leveraging digital and telehealth strategies can extend integrated neuropsychiatric care beyond the inpatient setting into outpatient follow-up and remote monitoring. In Latin America, telemedicine adoption has grown markedly since the COVID-19 pandemic, demonstrating feasibility for mental health interventions despite infrastructure challenges; pilot studies indicate reasonable acceptability but highlight the need for cost and outcome data in our region (26–29). Embedding teleconsultation for post-discharge psychiatric follow-up and tele-neurology evaluations could facilitate early identification of decompensation and timely interventions, potentially reducing readmissions.

Applying artificial intelligence (AI) and machine learning to multimodal data may help identify subgroups of patients who benefit most from specific interventions. For example, combining clinical scales, neuroimaging connectivity metrics, EEG features, and biomarker profiles could support predictive models of treatment response or risk of relapse. Early work in precision psychiatry underscores the potential of ML for prognosis and personalized treatment, though challenges remain in obtaining sufficiently large, diverse datasets (30). A future direction is to build multicenter collaborations to pool data across Latin America, increasing sample size and representativeness, and to adopt explainable AI methods to ensure clinical interpretability.

Emphasizing equity and access is crucial. Latin American populations often face disparities in access to specialized neuropsychiatric services. Future directions include community outreach and partnership with regional centers to develop hub-and-spoke models, where ENPQ specialists provide remote support and training to peripheral hospitals. Research should examine barriers (e.g., digital divide, socioeconomic factors) and tailor interventions to underserved groups, ensuring inclusive implementation of integrated care (29).

Training and workforce development must parallel service expansion. Designing a structured fellowship curriculum, with defined competencies in both neurology and psychiatry, rotations in EEG labs, psychopharmacology seminars, and research methodology modules, will cultivate future neuropsychiatrists. Leveraging models from established programs (e.g., Stanford, Massachusetts General Hospital) and adapting them to local context can strengthen training pipelines in Brazil and Latin America.

## 5 Conclusion

Our retrospective evaluation of the first 26 admissions to the ENPQ demonstrates that an integrated neuropsychiatry ward is feasible in a Brazilian quaternary public hospital and yields encouraging initial outcomes. Our population had a mean age of 48.0 ± 15.4 years with 69.2% female, reflecting the referral profile; the mean length of stay was 26.1 days, longer than the 16.7 days reported in a large Brazilian tertiary neurology ward cohort but in line with expectations for severe, complex cases requiring simultaneous neurologic and psychiatric management. Importantly, the 30-day readmission rate was 7.6%, notably lower than the approximately 12.5% seen in non-integrated neurology wards and the 10–20% (pooled ~16%) reported for general psychiatric wards (23).

These findings suggest that concurrent management of overlapping neurological and psychiatric needs may enhance stabilization before discharge. However, the small sample size, retrospective design, absence of a control group, and potential referral biases limit generalizability. Nonetheless, the observed outcomes provide preliminary evidence that integrated care can achieve comparable or better short-term stability in a complex patient population. Future prospective controlled studies with larger cohorts are necessary to confirm these results and determine whether integrated neuropsychiatric wards consistently reduce readmissions and optimize resource use.

## Data Availability

The original contributions presented in the study are included in the article/supplementary material. Further inquiries can be directed to the corresponding author.
